# Metabolomic Profiling of Serum Reveals Energy Metabolism Differences in Nellore Bulls with Divergent Growth Rates during Feedlot Finishing

**DOI:** 10.1021/acsomega.5c05181

**Published:** 2025-10-06

**Authors:** José B. S. Moreira, Richard V. Ribeiro, Nara R. B. Cônsolo, Gabriel H. Ribeiro, Luiz A. Colnago, Rodrigo N. S. Torres, Otávio R. Machado Neto, Rogério A. Curi, Luis Artur L. Chardulo, Welder A. Baldassini

**Affiliations:** † School of Agricultural and Veterinary Sciences, 28108 São Paulo State University (UNESP), Jaboticabal, São Paulo 14884-900, Brazil; ‡ School of Veterinary Medicine and Animal Science, 28133 University of São Paulo (USP), Pirassununga, São Paulo 13635-900, Brazil; § 564899 EMBRAPA Instrumentação, São Carlos, São Paulo 13560-970, Brazil; ∥ School of Veterinary Medicine and Animal Science, São Paulo State University (UNESP), Botucatu, São Paulo 18618-681, Brazil

## Abstract

This study aimed to identify and quantify serum metabolites in beef cattle exhibiting different growth rates during the finishing phase. A total of 120 Nellore (*Bos indicus*) bulls, averaging 387 ± 14 kg in body weight and 24 ± 2 months of age, were evaluated. The animals were housed for 115 days, and on day 21 (end of the first adaptation step), blood samples were collected from the coccygeal vein for metabolomic analysis. Based on average daily gain (ADG), two contrasting groups were selected: high performance (HP; *n* = 12) and low performance (LP; *n* = 12). Serum samples collected on day 21 were analyzed by proton nuclear magnetic resonance (^1^H NMR) to extract and quantify metabolites. Longissimus muscle area (LMA), backfat thickness (BFT), and hot carcass weight (HCW) were measured via ultrasound at the end of the finishing period. Animal performance was affected by growth rate, with HP animals showing significantly greater final body weight, HCW, and BFT (*p* < 0.05). A total of 47 serum metabolites were identified, including essential and nonessential amino acids, sugars, peptides, vitamins, amino acid derivatives, and organic acids. HP cattle exhibited higher concentrations of threonine, glycolate, ornithine, histidine, and creatinine (*p* < 0.05), while LP animals showed greater levels of phenylalanine, succinate, acetate, asparagine, and 2-hydroxyisobutyrate (*p* < 0.05). Key enriched pathways included the mitochondrial electron transport chain (*p* = 0.06), ethanol degradation (*p* = 0.08), and threonine and 2-oxobutanoate degradation (*p* = 0.09). These findings suggest enhanced energy metabolism in HP animals, driven by greater substrate diversity, while LP animals may exhibit impaired mitochondrial function, negatively impacting performance.

## Graphical abstract

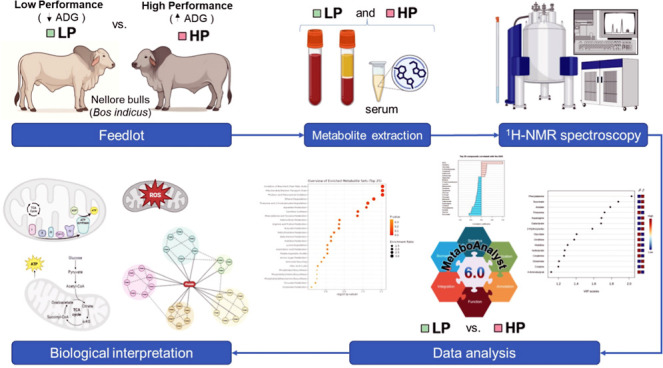

## Introduction

With the growing global demand for food, there is an urgent need to adopt strategies that enhance meat production without expanding agricultural land or contributing to deforestation in animal production systems.^1^ Feedlot finishing has emerged as a widely adopted approach to increase beef output while alleviating pressure on forested areas, as it supports improved animal performance.^2^ While this system delivers significant productivity gains, it also presents metabolic challenges, particularly for breeds adapted to tropical climates, such as Nellore cattle.^3^ The shift from pasture-based to high-energy feedlot diets necessitates metabolic adaptation, which is essential for optimizing productivity.^4^

Beyond environmental factors, cattle performance is also shaped by individual metabolic variability, which influences growth rates and directly affects the sustainability of intensive production systems.^5,6^ Average daily gain (ADG) is a primary metric used to assess cattle growth and is a vital tool for strategic decision-making in animal production.^7,8^ Variations in ADG reflect differences in nutrient utilization efficiency and the regulation of metabolic pathways among animals of the same breed, leading to distinct physiological profiles and body composition characteristics.^9^

To better understand the underlying causes of performance variation, it is crucial to investigate how metabolism is modulated in animals exhibiting superior growth, thereby enabling the identification of metabolic factors linked to growth and feed efficiency. Studies involving *Bos taurus* × *Bos indicus* crossbred steers with differing ADG have shown that animals with higher ADG exhibit altered metabolic activity, particularly in energy-related pathways such as β-oxidation.^10,11^ These animals displayed enhanced mobilization and oxidation of fatty acids, thereby increasing energy availability to support growth. However, findings in the literature are not entirely consistent regarding the influence of growth rate on metabolism. For instance, a study evaluating the metabolome of Charolais × Angus crossbred steers reported that animals with lower growth rates showed greater activity in oxidative metabolism pathways, while those with higher growth rates exhibited increased glycolytic and protein metabolism activity.^6^

Interactions between growth rate and metabolic profiles have been previously described in beef cattle.^10,12^ However, most of these studies have focused on assessments conducted at the end of the finishing phase, without addressing the initial metabolic status and its impact on subsequent performance.^6,10,12,13^ Identifying early metabolic signatures associated with later growth performance represents a promising strategy, as it may allow for more precise nutritional interventions aimed at enhancing ADG and overall productivity in beef cattle.

Recent studies have demonstrated the potential of metabolomics to assess the effects of production conditions on the metabolome of beef cattle.^14–16^ This analytical approach enables the characterization of animals’ metabolic profiles using tissues or biological fluids, facilitating the identification and quantification of metabolites involved in key metabolic pathways. Such insights form a foundation for understanding physiological responses related to production and for enhancing both productivity and efficiency.^17^ However, significant knowledge gaps remain regarding metabolic changes in Nellore cattle, particularly in relation to how growth rate influences their metabolome. In this context, the objective of the present study was to evaluate and identify alterations in the blood metabolic profile of Nellore cattle with differing ADG during the finishing phase.

## Results

### Performance

Table 2 presents the performance and carcass traits of the 40 animals that underwent blood sampling. From these 40 animals, 24 were selected for metabolomic analyses (12 with the greatest ADG and 12 with the lowest ADG), and their performance and carcass traits are shown in Table 3. In this group, no significant differences (*p* > 0.05) were observed between treatments for LMA. A trend was noted for dry matter intake (DMI) (*p* < 0.10). However, high-performance (HP) animals showed significant differences (*p* < 0.05) in final body weight (FBW), ADG, BFT, and HCW compared to low-performance (LP) animals, with higher values for all traits observed in the HP group (Table 3).

**Table 1:** Ingredients and Nutritional Composition of Experimental Diet

	adaptation				
	step 1	step 2	step 3	step 4	finishing
days on feed	21	4	4	4	82
Diet Ingredients (%)					
corn silage	24.97				
sugar cane bagasse	15.6	23.54	18.52	13.38	12.00
ground corn	18.31	28.40	38.70	50.74	51.07
citrus pulp	12.30	20.01	17.35	13.06	15.91
dried distiller grain	25.43	25.27	22.41	19.43	17.63
mineral–vitamin premix^a^	3.39	2.78	3.02	3.39	3.39
Diet Composition (%)					
dry matter	68.69	67.00	67.00	67.00	67.00
ashes	6.92	6.22	5.98	5.81	5.79
crude protein	14.50	14.30	14.00	13.75	13.50
ether extract	4.28	4.30	4.30	4.30	4.29
neutral detergent fiber	36.31	33.97	29.65	25.20	21.89
acid detergent fiber	23.48	17.75	15.06	12.28	10.50

a Composition (dry matter basis): 15% Ca; 1.9% S; 1.5% Mg; 4.5% Na; 1.6% P; 1.715 ppm Zn; 1.285 ppm Mn; 428 ppm of Cu; 21 ppm I; 5.7 ppm of Se; 8.5 ppm of Co; 285 ppm Fe; 86.000 IU vitamin A; 115.000 IU vitamin D_3_; 105 IU vitamin E; 17% urea.

**Table 2:** Mean, Standard Deviation (SD) and Minimum and Maximum Values of the Main Performance and Carcass Traits of Feedlot Nellore Bulls (*n* = 40)

variables^a^	mean	SD	minimum	maximum
FBW, kg	571.01	34.94	516.00	620.00
ADG, kg/day	1.58	0.25	1.17	1.94
DMI, kg/day	9.95	0.56	9.17	11.04
HCW, kg	317.67	17.78	291.00	355.00
LMA, cm^2^	72.87	7.68	59.20	88.70
BFT, mm	4.05	1.06	1.90	6.00

a Variables of 40 animals (2 per pen) randomly selected for blood collection. FBW: final body weight; ADG: average daily gain; DMI: dry matter intake; HCW: hot carcass weight; LMA: longissimus muscle area; BFT: backfat thickness.

**Table 3:** Performance and Carcass Traits of Nellore Bulls with High (HP) and Low (LP) Growth Rates during the Finishing Phase^b^

	treatment			
variable^a^	HP (*n* = 12)	LP (*n* = 12)	SEM	*P*-value
FBW, kg	602.17	540.00	4.30	<0.01
ADG, kg/day	1.79	1.36	0.03	<0.01
DMI, kg/day	10.16	9.75	0.15	0.07
HCW, kg	332.25	303.08	2.87	<0.01
LMA, cm^2^	73.93	71.82	2.24	0.51
BFT, mm	4.48	3.63	0.29	0.05

a Variables of 24 animals selected for metabolomic analyses. FBW: final body weight; ADG: average daily gain; DMI: dry matter intake; HCW: hot carcass weight; LMA: longissimus muscle area; BFT: backfat thickness.

b HP: high performance; LP: low performance.

### Metabolomic

A representative ^1^H NMR spectrum of the filtered serum, along with a detailed peak including chemical shifts (ppm) and signal multiplicities (e.g., singlet, doublet), is provided as Supporting Information (Table S1). A total of 47 metabolites were identified in the serum of the animals, including essential and nonessential amino acids, sugars, peptides, vitamins, amino acid derivatives, organic acids, and other compounds. No significant differences (*p* > 0.05) in metabolite concentrations were observed between treatments in the univariate analysis (Table 4).

**Table 4:** Metabolites Concentrations (mM) on Nellore Bulls Blood Serum with High (HP) and Low (LP) Growth Rates during the Finishing Phase

	treatment^a^			
metabolites	LP	HP	SEM^b^	*P*-value
1,3-dimethylurate	0.003	0.002	0.0003	0.863
2-hydroxyisobutyrate	0.005	0.004	0.0007	0.980
3-hydroxybutyrate	0.130	0.116	0.0110	0.501
3-hydroxyisovalerate	0.031	0.030	0.0030	0.772
3-phenylpropionate	0.006	0.006	0.0004	0.811
4-aminobutyrate	0.032	0.031	0.0010	0.471
acetate	0.198	0.169	0.0250	0.135
alanine	0.224	0.214	0.0120	0.572
allantoin	0.034	0.041	0.0050	0.776
asparagine	0.024	0.021	0.0010	0.084
benzoate	0.006	0.005	0.0003	0.419
betaine	0.064	0.053	0.0060	0.268
butyrate	0.008	0.007	0.0007	0.613
choline	0.009	0.009	0.0009	0.678
citrate	0.207	0.201	0.0130	0.613
creatine	0.110	0.083	0.0110	0.083
creatine phosphate	0.071	0.067	0.0030	0.656
creatinine	0.049	0.053	0.0030	0.279
dimethyl sulfone	0.013	0.011	0.0020	0.613
dimethylamine	0.003	0.002	0.0003	0.863
ethylene glycol	0.071	0.065	0.0070	0.593
formate	0.023	0.024	0.0020	0.306
galactarate	0.001	0.001	0.0003	0.957
glucose	3.716	3.071	0.2610	0.138
glutamate	0.025	0.026	0.0010	0.494
glutamine	0.145	0.145	0.0110	0.963
glycerol	0.143	0.129	0.0120	0.397
glycine	0.309	0.274	0.0170	0.301
glycolate	0.033	0.039	0.0030	0.271
hippurate	0.0129	0.0123	0.0010	0.861
histidine	0.025	0.027	0.0010	0.468
isobutyrate	0.011	0.011	0.0007	0.691
isoleucine	0.057	0.054	0.0030	0.606
lactate	6.161	5.271	1.0170	0.867
leucine	0.098	0.093	0.0050	0.458
mannose	0.042	0.038	0.0010	0.067
methanol	0.003	0.003	0.0003	0.186
methionine	0.014	0.013	0.0009	0.683
methylmalonate	0.027	0.025	0.0050	0.907
ornithine	0.021	0.023	0.0010	0.214
phenylalanine	0.0225	0.021	0.0009	0.287
proline	0.049	0.047	0.0020	0.318
pyruvate	0.005	0.005	0.0005	0.789
succinate	0.004	0.004	0.0009	0.613
threonine	0.032	0.029	0.0030	0.614
tyrosine	0.055	0.053	0.0030	0.731
valine	0.159	0.149	0.0090	0.473

a HP: high performance; LP: low performance.

b SEM: standard error of the mean.

PLS-DA was employed to visualize the separation between the analyzed groups. Although components 1 and 2 explained a relatively small portion of the variance (cumulative variance of 14.5%), the analysis indicated differences between the experimental groups (HP and LP), as shown by the nonoverlapping ellipses (Figure 1). The PLS-DA model effectively captured relevant distinctions between the groups (*R* ^2^ = 0.91).

**Figure 1:**
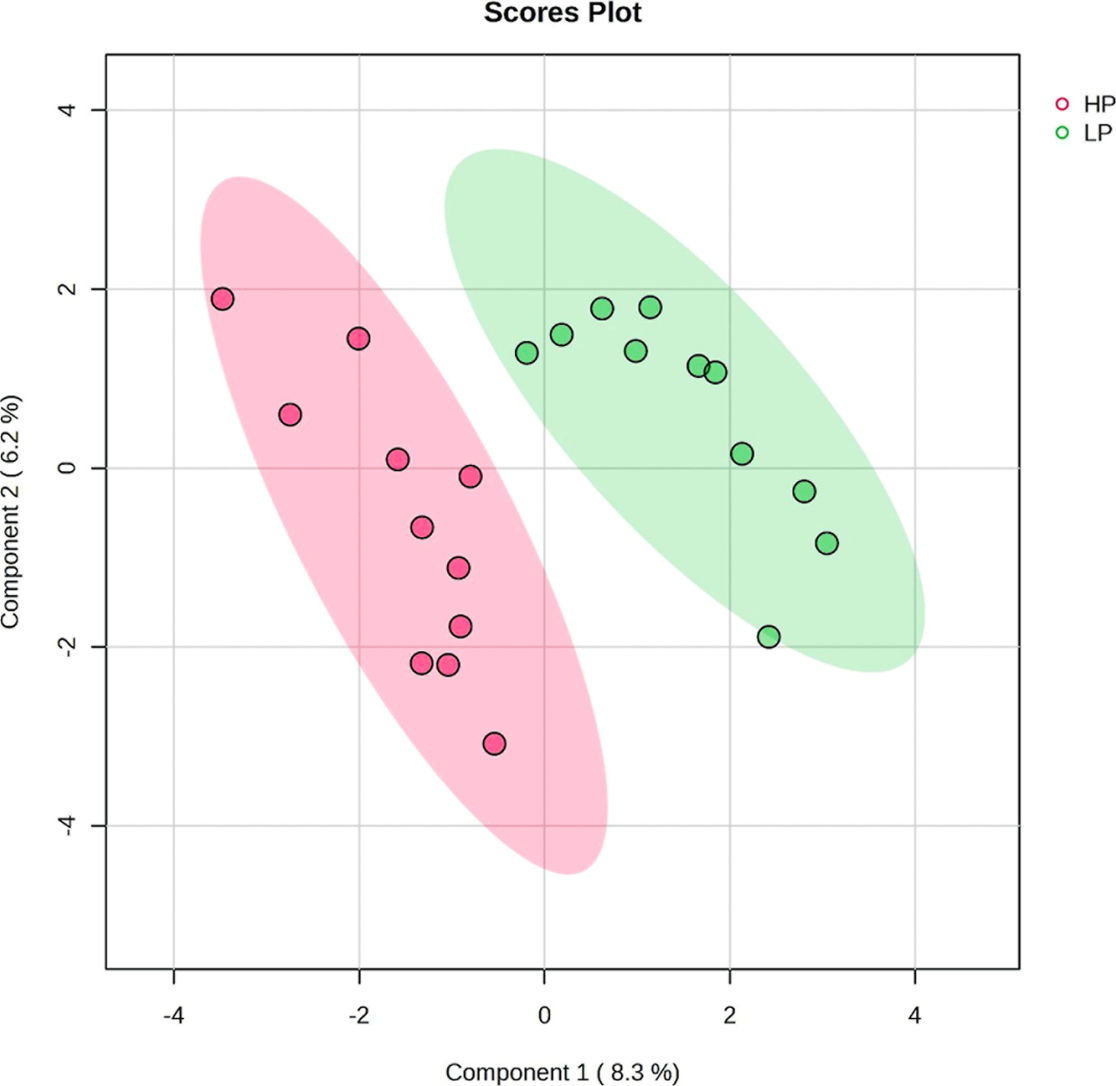
Partial least squares discriminant analysis (PLS-DA) of the serum metabolomic profiles of Nellore bulls with differing growth rates during feedlot finishing. HP = high performance; LP = low performance.

Considering the distinction between the HP and LP groups demonstrated by the PLS-DA, a variable importance in projection (VIP) analysis was performed, identifying 15 metabolites contributing to the separation between the experimental groups (Figure 2). A cutoff value of 1.0 was applied to determine the most influential metabolites. The HP group exhibited higher VIP scores for threonine, glycolate, histidine, isobutyrate, and creatinine, while the LP group showed higher scores for phenylalanine, succinate, acetate, asparagine, galactarate, and 2-hydroxyisobutyrate (2-HIB). Since multivariate analysis is influenced by the integration of multiple metabolites, it is not unusual to observe divergences between univariate and multivariate results, a pattern also observed in this study.

**Figure 2:**
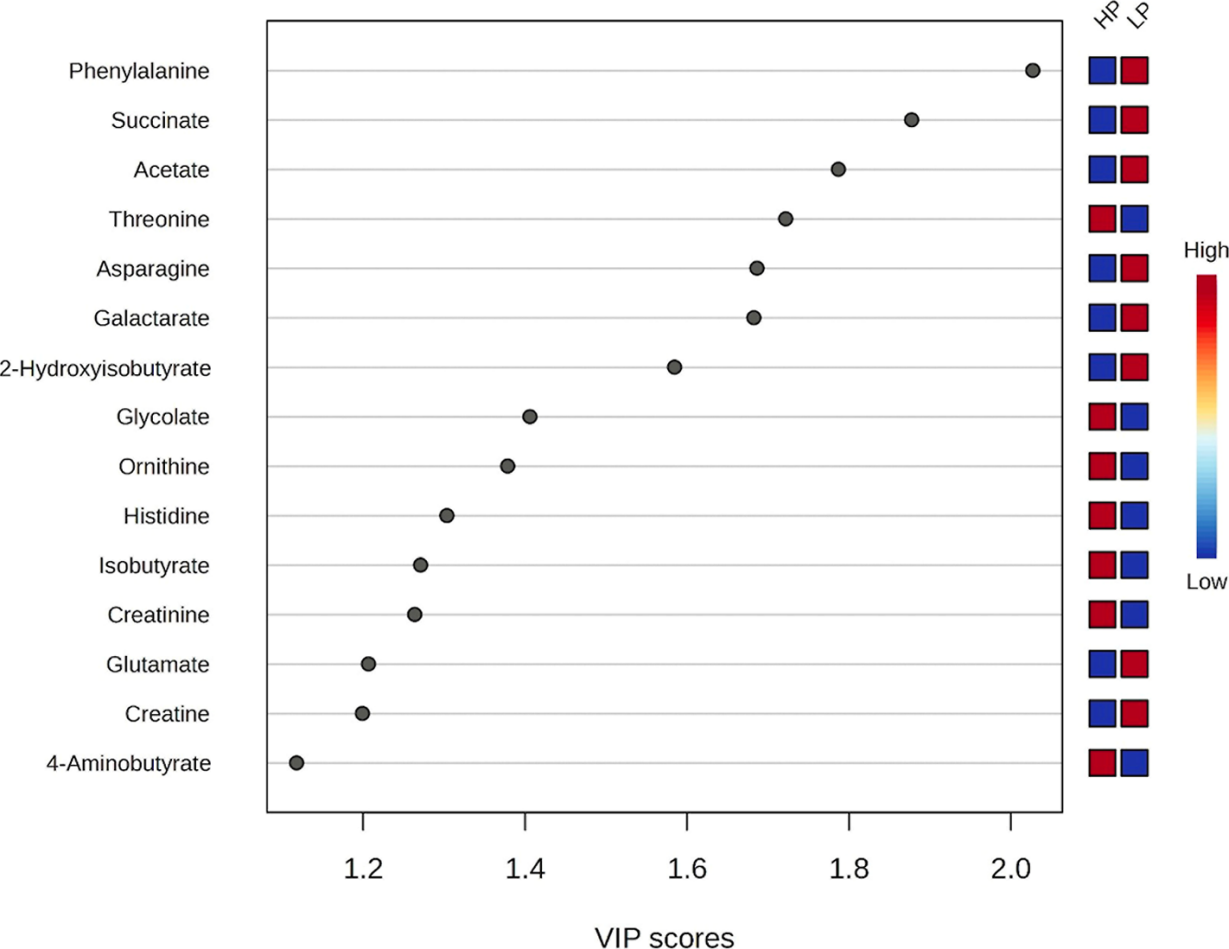
Variable importance in projection (VIP) scores for serum metabolites in Nellore bulls with differing growth rates during the finishing phase. HP = high performance; LP = low performance.

Metabolic pathways that differed between the HP and LP groups were also identified (Figure 3). The most affected pathways included oxidation of branched-chain fatty acids (*p* = 0.06), mitochondrial electron transport chain (*p* = 0.06), peroxisomal oxidation of phytanic acid (*p* = 0.06), ethanol degradation (*p* = 0.08), and degradation of threonine and 2-oxobutanoate (*p* = 0.09). Additionally, correlations were observed between the identified metabolites and ADG, with acetate, succinate, and glycine showing negative associations (Figure 4).

**Figure 3:**
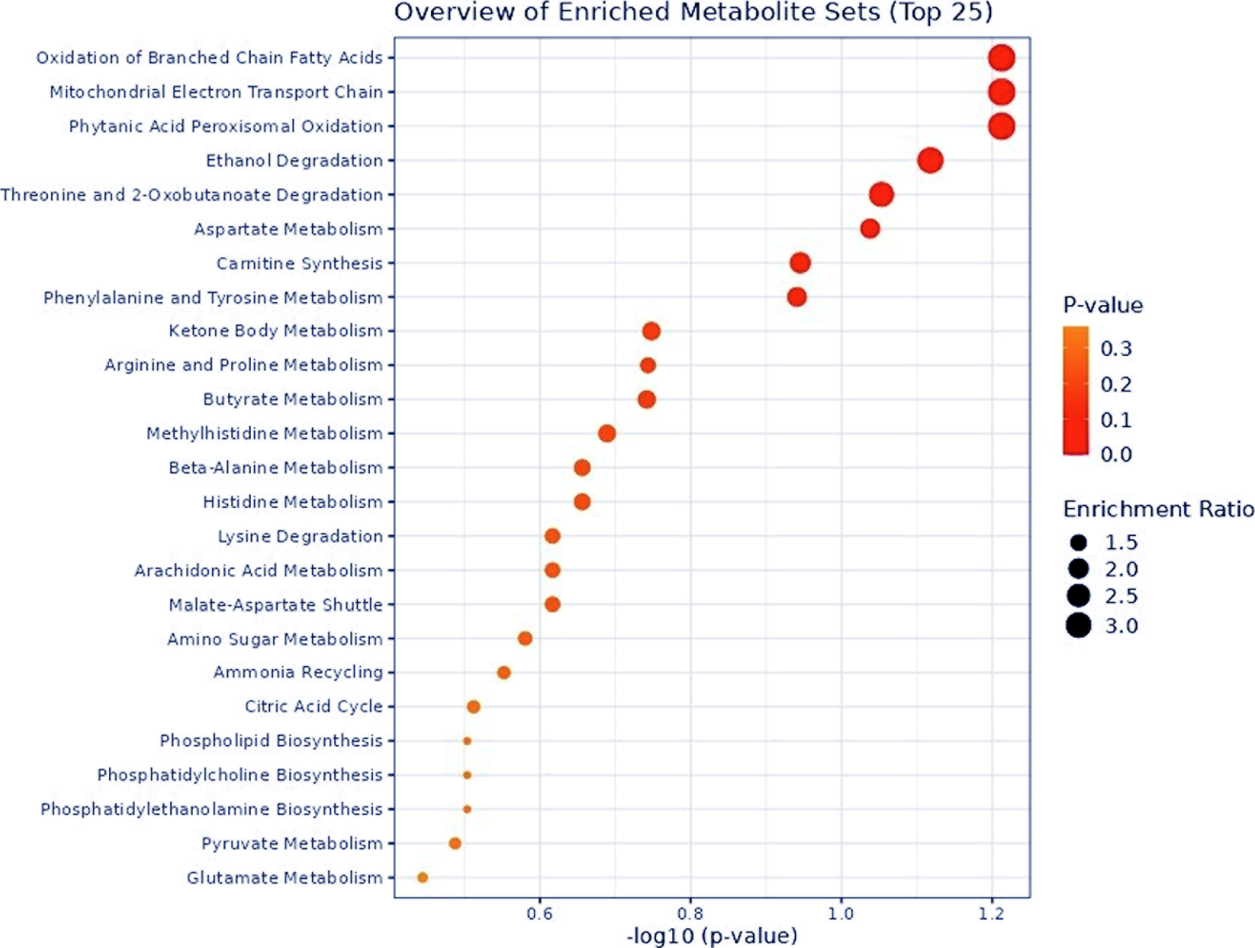
Metabolic pathway enrichment analysis of serum samples from Nellore bulls with differing growth rates during the finishing phase. HP = high performance; LP = low performance.

**Figure 4:**
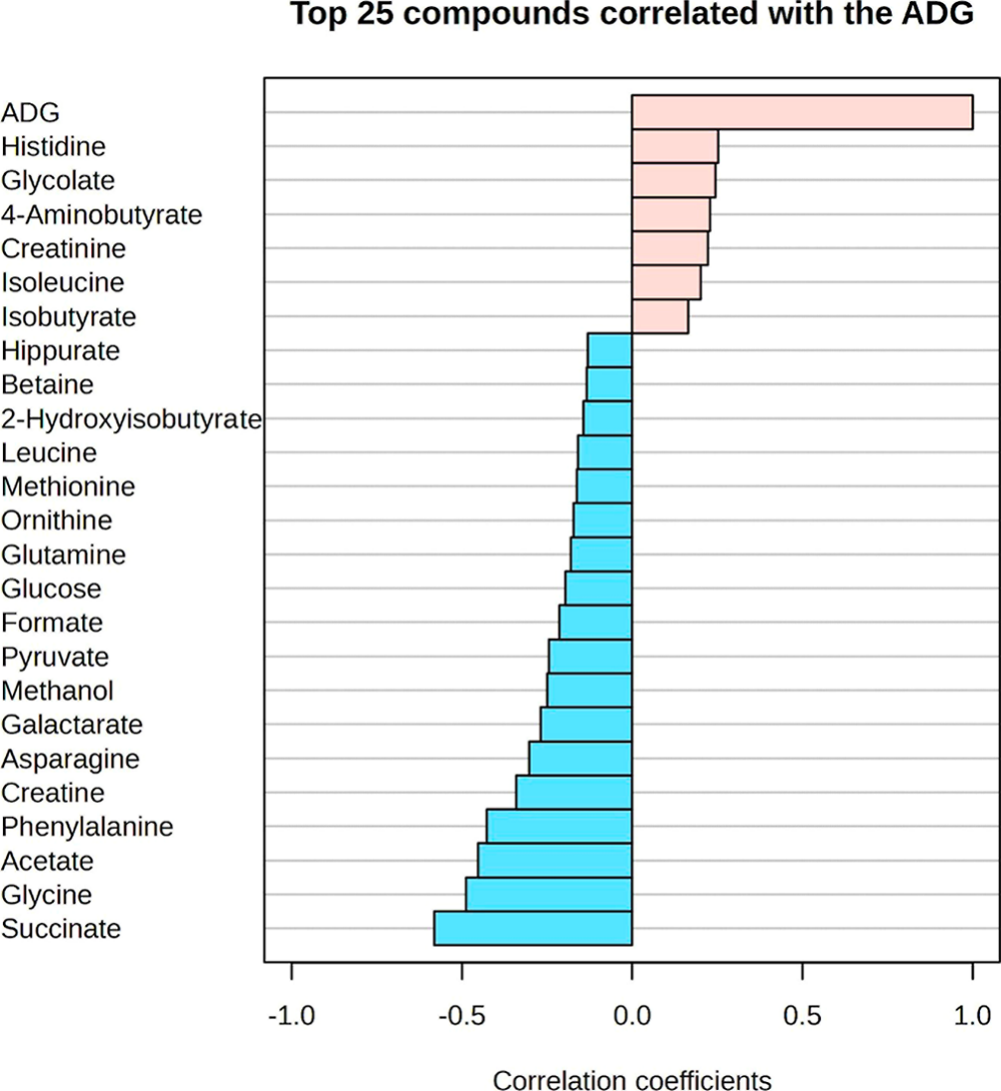
Correlation between serum metabolites and average daily gain (ADG) in Nellore bulls with differing growth rates during the finishing phase. HP = high performance; LP = low performance.

## Discussion

Identifying the factors that influence animal performance is crucial for enhancing productivity in the beef industry. Animals with higher ADG (HP) demonstrated superior performance during the finishing phase, exhibiting greater FBW, HCW, and BFT, while showing similar LMA values compared to LP animals. Additionally, HP cattle tend to produce heavier carcasses, a result of the cumulative effect of increased weight gain.^18,19^ The observed tendency in DMI between performance groups is expected, as DMI is the main factor influencing variations in ADG, providing a greater amount of nutrients available for digestion.^20^ This outcome is also closely associated with improved feed efficiency, as high-performing animals exhibit physiological adaptations in the gut that enhance nutrient utilization, ultimately supporting growth and overall performance.^9^ Previous studies have reported that crossbred steers (Angus × Hereford) with varying growth potentials exhibited a similar pattern, with higher FBW, HCW, and BFT values observed in animals with greater growth potential and no significant differences in LMA or visceral fat.^21^ The authors attributed the increase in HCW to the higher FBW, as no increase in LMA was detected—a finding consistent with the present study.

Given that blood transports metabolic products from tissues and organs and shows a strong correlation with molecules found in other biological samples, the metabolites identified in this study may offer valuable insights into the overall metabolism of cattle with differing growth rates, as supported by previous research.^22,23^ Furthermore, a study analyzing the metabolic profiles of serum, feces, and urine from dairy cows reported a positive correlation among metabolites across these biological matrices, with approximately 75% of the metabolites detected in blood also present in the other samples.^24^

In the current study, the metabolites with higher VIP scores distinguishing animals with varying growth rates were primarily associated with energy and protein metabolism, including phenylalanine, threonine, succinate, and acetate. These findings underscore metabolic differences relevant to weight gain optimization. This outcome may be linked to the fact that protein synthesis is an energy-demanding process, requiring substantial energy efficiency in growing animals.^25^ Unlike nonruminants, ruminants have limited glucose availability in the intestine and depend largely on gluconeogenesis for glucose synthesis, as well as on alternative metabolic pathways for energy production.^26,27^ A study conducted in Dorset sheep found that ruminants facing high energy demands expand their use of energy substrates by increasing amino acid degradation for gluconeogenesis.^28^ In line with this, cattle in the HP group exhibited elevated concentrations of metabolites involved in energy metabolism, particularly those related to gluconeogenesis, the urea cycle, and mitochondrial function.

Among the metabolites with the highest concentrations in the blood of HP cattle and with high VIP scores for group separation was threonine, an amino acid with important roles in lipid metabolism, protein synthesis, and intestinal health and function.^29^ Additionally, threonine can contribute to gluconeogenesis, acting as one of the amino acids that donate carbon to this pathway.^30^ The significance of threonine in ruminant metabolism was demonstrated in studies on castrated sheep, where carbon transfer from threonine to glucose increased in animals with greater energy demands.^31^ A similar result was found when associating performance traits and various metabolites in the plasma of crossbred beef cattle (*B. taurus* × *B. indicus*), where higher threonine concentrations were observed in animals with greater feed efficiency.^32^

Higher concentrations of histidine were also detected in HP animals. Like threonine, histidine is a glucogenic amino acid and is considered the primary growth-limiting amino acid in cattle.^30,33,34^ A study in lactating cows with high energy demands showed that increasing histidine supplementation led to greater hepatic glucose production and upregulation of genes involved in gluconeogenesis.^35^ The elevated concentrations of glucogenic amino acids in HP cattle may reflect a diversification of substrate use for energy production in ruminants under high energy demand. Functional enrichment analysis revealed that the degradation pathways of threonine and 2-oxobutanoate, histidine metabolism, and the TCA cycle were enriched—supporting the hypothesis that amino acids such as threonine and histidine are catabolized for energy production, entering the TCA cycle via conversion to succinyl-CoA.^36,37^

Ornithine is a nonproteinogenic amino acid that plays a crucial role in the urea cycle, aiding in the removal of excess ammonia produced during the metabolism of nitrogenous compounds, such as amino acids.^38^ The higher concentration of ornithine observed in HP animals may suggest enhanced regulation of the urea cycle, likely driven by increased amino acid catabolism for energy production—particularly since hepatic ammonia accumulation can impair gluconeogenesis.^28^ Similarly, a greater abundance of enzymes involved in gluconeogenesis has been reported in Angus × Nellore crossbred cattle.^39^

The elevated concentration of creatinine in the serum of HP animals at the onset of the feedlot finishing phase may reflect their higher energy demands compared to LP animals, as creatinine is involved in cellular energy generation.^40^ A key component of muscle energy metabolism, creatinine is formed from the breakdown of phosphocreatine, a process that rapidly produces ATP.^41^ Increased creatinine levels have also been documented in fast-growing animals, highlighting the importance of efficient, rapid energy generation in supporting their growth.^6^

Higher concentrations of free amino acids, such as phenylalanine and asparagine, in the serum of LP cattle may indicate reduced efficiency in utilizing available amino acids for protein synthesis. This inefficiency likely leads to the accumulation of these metabolites in the bloodstream. Phenylalanine is an essential and limiting amino acid in cattle and plays a key role in the synthesis of other amino acids, such as tyrosine.^42,43^ Elevated phenylalanine levels have also been observed in the plasma of inefficient cattle with high residual feed intake (RFI).^32^ Despite these differences in protein metabolism between HP and LP groups, no effect on LMA was detected, aligning with previous studies in Nellore bulls divergent for RFI.^44^

Differences in energy metabolism between LP and HP cattle were also evident in pathways involving succinate and acetate. These metabolites participate in distinct metabolic routes, typically being converted into succinyl-CoA and acetyl-CoA, respectively, and entering the TCA cycle for energy production.^45^ The accumulation of these intermediates in slow-growing animals may reflect reduced energy efficiency associated with TCA cycle, indicating suboptimal utilization of available substrates. Similar trends have been reported, with higher concentrations of acetate and succinate found in inefficient (high RFI) animals.^32^

Acetate is one of the primary precursors of lipid synthesis in ruminants, being converted into acetyl-CoA and subsequently into malonyl-CoA, which serves as the foundational structure for fatty acid synthesis.^46^ Moreover, the acyl group derived from acetate contributes approximately 70–80% of the total acyl units required for subcutaneous fat deposition in cattle.^47^ The lower BFT observed in LP animals may be linked to their elevated serum acetate concentrations, potentially reflecting inefficiencies in converting acetate into fatty acids and thereby impairing lipogenesis. A similar association has been reported, with a negative correlation observed between plasma acetate levels and BFT at slaughter in crossbred Wagyu steers.^48^

The higher concentration of succinate observed in LP cattle may be linked to increased oxidation via succinate dehydrogenase, resulting in greater electron flux through the mitochondrial electron transport chain (ETC). This excess flux may be dissipated as heat, reducing energy efficiency and promoting the generation of reactive oxygen species (ROS).^49^ The accumulation of ROS is harmful, as it is associated with lipid peroxidation particularly of polyunsaturated fatty acids—and may trigger various forms of cell death, such as apoptosis and autophagy, ultimately raising the animal’s maintenance energy requirements.^50,51^ Furthermore, excessive ROS in inefficient animals may stimulate the ubiquitin–proteasome system and activate the Akt/mTOR signaling pathway, promoting protein degradation and inhibiting protein synthesis, as shown in studies on cattle with divergent feed efficiency.^52^

This hypothesis is further supported by the enrichment of mitochondrial ETC and ethanol degradation pathways, the latter of which produces acetate.^53^ The negative correlations observed between succinate and acetate with ADG in this study reinforce the idea that the accumulation of these metabolites adversely affects performance during the finishing phase. Research in cattle and sheep has suggested that mitochondrial ETC inefficiency may be a contributing factor to poor energy efficiency in animals with low weight gain and high RFI or overall low feed efficiency.^54,55^ However, this relationship remains complex, as contradictory findings have also been reported, with higher succinate concentrations found in the muscle and ruminal fluid of more efficient animals.^12,56^

LP animals exhibited higher serum concentrations of 2-hydroxyisobutyrate (2-HIB) compared to HP animals. This compound is a secondary metabolite of valine metabolism, although its exact physiological role remains unclear.^57^ Studies in young rats have identified 2-HIB as a stress marker, showing elevated plasma levels and reduced hepatic concentrations in animals experiencing growth restriction and diminished protein deposition.^58^ Other studies have linked 2-HIB to glucose metabolism, with increased levels observed in individuals with diabetes mellitus and in undernourished rats, suggesting a possible connection to impaired insulin activity.^59,60^ This may reflect reduced insulin sensitivity in LP cattle, a trend also reported in low-ADG Angus calves.^61^ Additionally, 2-HIB has been associated with ethanol intake in both rats and humans,^62,63^ and its higher concentration in LP animals may be related to ethanol degradation, a pathway enriched in this study.

Despite the insights gained from the present study, some limitations should be acknowledged. Complementary physiological or biochemical markers (e.g., insulin, cortisol, liver enzymes) were not evaluated, which limits the integration of metabolomic data with additional indicators of metabolic status. Additionally, detailed genetic information was not available, as the animals were acquired from a commercial farm. Nevertheless, all animals were purebred Nellore and belonged to a single contemporary group, which helps to minimize potential sources of genetic and management-related variability. Together, these factors should be considered when interpreting the results and represent important directions for future research.

Overall, the findings suggest that feedlot performance in Nellore bulls is influenced not only by growth traits but also by their inherent ability to regulate metabolic responses under production conditions. Understanding how animal metabolism shapes performance, and identifying which aspects of the process are most closely linked to inefficiency, can provide key information for the development of targeted technologies. The differences in metabolomic profiles observed between HP and LP animals may provide support for the future establishment of biomarkers to identify animals with superior growth performance at an earlier stage, although further validation is needed.

## Conclusion

The results of this study indicate that Nellore bulls with divergent growth rates exhibit distinct metabolic adaptations during the feedlot period. Animals with higher ADG demonstrated a metabolic profile indicative of greater energy and protein efficiency, reflected by elevated concentrations of metabolites involved in gluconeogenesis, the urea cycle, and mitochondrial function, such as threonine, histidine, and ornithine. In contrast, bulls with lower ADG showed an accumulation of metabolites like succinate and acetate, suggesting decreased efficiency in utilizing energy substrates. These metabolic distinctions underscore that feedlot performance is closely tied to an animals’ innate capacity to modulate metabolism in response to the energy demands of growth. Accordingly, identifying these metabolic biomarkers may inform the development of targeted nutritional and genetic selection strategies to enhance productive efficiency in *B. indicus* cattle.

## Materials and Methods

All animal procedures and biological sampling in this study were approved by the Ethics Committee on the Use of Animals (CEUA) at the School of Veterinary Medicine and Animal Science–FMVZ, UNESP Botucatu (Protocol No. 0585/2023).

### Location, Animals, and Treatments

A total of 120 Nellore (*B. indicus*) bulls, with an average initial body weight of 387 ± 14 kg and an age of 24 ± 2 months, were enrolled in the study. During the adaptation period, five animals were removed due to difficulties in adjusting to the diet, resulting in a final cohort of 115 animals. The animals were housed in a covered shed with collective concrete pens measuring 30 m^2^, with five animals per pen. They were obtained from a commercial farm in the region and had been treated for both endo- and ecto-parasites at the source. Before the experiment began, the cattle underwent a seven-day acclimation period in the feedlot, during which they were fed corn silage to aid recovery from transport-related stress.

After this period, the animals were weighed and began a gradual adaptation to the finishing diet following a four-step protocol. The first step lasted 21 days, during which the animals received a diet containing 40% roughage. In the subsequent phases, the grain proportion was gradually increased, with dietary changes implemented every 4 days. At the end of the first phase, the animals were weighed again, and blood samples were collected. They were fed twice daily, at 09:00 and 16:00 h. The estimated composition and formulation of the diet (Table 1) were generated using the MAX System for Beef software (Cargill Incorporated, Wayzata, Minnesota, United States).

### Blood Sampling, Performance and Experimental Groups

Each day, the dry matter offered and orts per pen were recorded to estimate DMI. On day 21, marking the end of the first adaptation phase, two animals per pen (*n* = 40) were randomly selected for blood sampling. Samples were collected from the coccygeal vein using vacuum collection tubes (Vacutube, Biocon Diagnostics, Belo Horizonte, Minas Gerais, Brazil) containing a clot activator. Samples were immediately centrifuged (2000*g* for 15 min), and the resulting serum was stored at −80 °C in polypropylene tubes (Eppendorf Safe-Lock, Eppendorf, Hamburg, Germany). At the conclusion of the finishing period, the animals were reweighed to determine FBW, underwent ultrasound evaluation (Esaote Pie Medical, Pie Medical Equipment B.V., Maastricht, Limburg, The Netherlands) using a 3.5 MHz probe and Echo Image Viewer 1.0 software (Pie Medical Equipment B.V., Maastricht, Limburg, The Netherlands), and were subsequently sent to a commercial slaughterhouse.

Animal performance data were calculated using weight measurements and the number of days between them, applying the following formula:

Average daily gain

$$ADG=\frac{initial\;body\;weight-final\;body\;weight}{days\;between\;weighs}$$

Estimation of DMI

$$DMI=\frac{\frac{average\;DM\;offered-average\;DM\;orts}{days\;on\;feed}}{number\;of\;animals\;per\;pen}$$

The carcass traits assessed included hot carcass weight (HCW), measured by weighing the carcasses immediately postslaughter; longissimus muscle area (LMA) and backfat thickness (BFT), evaluated using ultrasound images of the *Longissimus thoracis* muscle between the 12th and 13th ribs.

At the end of the finishing period, from the subset of 40 animals sampled for blood collection, the 12 bulls with the highest ADG (high-performance group, HP) and the 12 bulls with the lowest ADG (low-performance group, LP) were selected for metabolomic analysis. Performance and carcass data from these 24 animals were also used to compare HP and LP groups. Serum samples from these animals were analyzed by proton nuclear magnetic resonance spectroscopy (^1^H NMR).

### Sample Preparation, Acquisition, and Processing of NMR Spectra

For metabolite extraction, serum samples were thawed at room temperature, and 500 μL were centrifuged through 3 kDa filters at 14,000*g* for 30 min at 4 °C. Subsequently, 300 μL of the filtrate were lyophilized using a vacuum centrifuge (UVS800DA, THERMO Savant, Sunnyvale, California, United States) and submitted for ^1^H NMR analysis at EMBRAPA Instrumentation in São Carlos, SP, Brazil. The lyophilized residues were reconstituted in 550 μL of phosphate buffer in 0.1 M D_2_O (pD = 7.4) containing 0.5 mM of 3-(trimethylsilyl)-1-propanesulfonic acid-*d*_6_ (DSS-*d*_6_; Cambridge Isotopes, Leicestershire, United Kingdom), used as an internal standard of known concentration for metabolite quantification. The solution was transferred to a standard 5 mm NMR tube for measurement.

Spectra were acquired using a Bruker Avance III 14.1 T spectrometer (Bruker Corporation, Karlsruhe, Baden-Württemberg, Germany) equipped with a 5 mm Broadband Observe (BBO) probe featuring ATMA (Automatic Tuning Matching Adjustment), a *z*-gradient coil, a BCU-I variable temperature unit, a gradient generator, and a SampleXpress automatic sample changer. Standard ^1^H spectra were recorded at 298.15 K using the NOESY-1D pulse sequence (noesypr1d in Bruker TopSpin software), with water signal suppression by irradiation at 2821.88 Hz (O1). Acquisition parameters were number of scans (ns) = 256, spectral width (sw) = 12,019 Hz (20.0276 ppm), 90° pulse length (P1) = 14.85 μs, acquisition time (aq) = 4.50 s, relaxation delay (d1) = 4 s, data points (TD) = 108,170 (106 K), mixing time (d8) = 5 ms, and dummy scans (ds) = 4.

^1^H NMR spectra were processed using a 0.3 Hz line broadening in TopSpin 3.6.1 software (Bruker Biospin, Ettlingen, Baden-Württemberg, Germany). Manual phase and baseline corrections were applied using Chenomx NMR Suite 8.4 (Chenomx Inc., Edmonton, Alberta, Canada). Metabolites were manually identified in 1D ^1^H NMR spectra with the aid of the compound library integrated into the Chenomx Profiler tool. The resulting metabolite concentration table (47 metabolites × 24 samples) was exported to Excel, where sample identifiers were subsequently added.

### Statistical and Bioinformatics Analyses

Performance and carcass trait data were analyzed using SAS software (Version 9.4, SAS Institute, Cary, New York, United States). Residual normality was assessed using the Shapiro–Wilk test, and outliers were removed via the UNIVARIATE procedure. Outliers were identified based on externally studentized residuals (module) > 2.5. Homogeneity of variances was evaluated using the Box–Cox test. For variables not normally distributed, data were transformed using the PROC RANK procedure (SAS 9.4). The data were analyzed in a completely randomized block design using the PROC MIXED, where the animal was the experimental unit, treatments were the fixed effects, and block and animals were random effects. Due to the difference found in the initial body weight, this variable was adopted as covariable for the model, defined as follows

$$Y_{ijt}=\mu +\beta \cdot {IBW}_{ijt}+B_{i}+A_{j}+Q_{t}+e_{ijt}$$

where *Y*_*ijt*_ represents the dependent variable, μ is the overall mean, β·IBW_*ijt*_ is the regression coefficient (β) associated with the covariate initial body weight (IBW), *B*_*i*_ corresponds to the random effect of the block, *A*_*j*_ to the random effect of the experimental unit, *Q*_*t*_ to the fixed effect of the treatment, and *e*_*ijt*_ to the residual error.

Results were reported as least-squares means (LSMEANS statement). When significant main effects were identified, a posthoc Tukey test was applied to assess differences between means. Differences were considered statistically significant at *p* < 0.05, and trends were noted when 0.05 ≤ *p* < 0.10.

Metabolomic data were analyzed using the MetaboAnalyst 6.0 platform (http://www.metaboanalyst.ca/). Metabolite concentration data were log-transformed and scaled using the Pareto method prior to analysis. To explore group separation, supervised analyses were performed using partial least squares discriminant analysis (PLS-DA), with leave-one-out cross-validation. Classification accuracy was used as the performance metric.^6^ VIP scores were used to rank metabolites based on their contribution to group discrimination, with VIP values greater than 1.0 indicating metabolites most relevant for separation.

The metabolomic data set was further analyzed using bioinformatics approaches, including Metabolite Set Enrichment Analysis, based on the metabolite profiles of each group.^64^ Compound names were standardized according to KEGG IDs, and pathway analysis was conducted using the global test and relative betweenness centrality algorithms, with the *B. taurus* library selected as the reference.

## Supplementary Material



## Notes

Table S1: assignments of the ^1^H NMR spectrum of metabolites identified in the serum samples in 0.1 mol L^–1^ phosphate buffer. Chemical shifts (in ppm), multiplicity, and coupling constants (in Hz) for hydrogens (PDF)
