# Supplementary material for: Metabolomic Profiling of Serum Reveals Energy Metabolism Differences in Nellore Bulls with Divergent Growth Rates during Feedlot Finishing
Source: ACS Omega. 2025 Oct 6;10(41):48272–81. doi: 10.1021/acsomega.5c05181 (PMC12547783; doi:10.1021/acsomega.5c05181)
Supplement: Supplementary file 1 [file ao5c05181_si_001.pdf]

METABOLOMIC PROFILING OF SERUM REVEALS ENERGY METABOLISM DIFFERENCES IN NELLORE BULLS WITH DIVERGENT GROWTH RATES DURING FEEDLOT FINISHING

José B. S. Moreira[1], Richard V. Ribeiro[1], Nara R. B. Cônsolo[2], Gabriel H. Ribeiro[3], Luiz A. Colnago[3], Rodrigo N. S. Torres[4], Otávio R. Machado Neto[1,4], Rogério A. Curi[1,4], Luis Artur L. Chardulo[1,4], Welder A. Baldassini[1,4*]

[1] School of Agricultural and Veterinary Sciences, São Paulo State University (UNESP), Jaboticabal (São Paulo), 14884-900, Brazil.

[2] School of Veterinary Medicine and Animal Science, University of São Paulo (USP), Pirassununga (São Paulo), 13635-900, Brazil.

[3] EMBRAPA Instrumentação, *São Carlos (São Paulo), 13560-970, Brazil.*

[4] School of Veterinary Medicine and Animal Science, São Paulo State University (UNESP), Botucatu (São Paulo), 18618-681, Brazil.

**Corresponding author: w.baldassini@unesp.br*

Supporting information

Table S1. Assignments of the ^{1}H NMR spectrum of the metabolites identified in the serum sample in 0.1 mol L^{-1} phosphate buffer. Chemical shifts (in ppm). multiplicity. coupling constants (in Hz) for hydrogens

**Metabolites**	**^{1}H**
1,3-Dimethylurate	3.43 s; 3.30 s; 3.30 s
3-Hydroxybutyrate	1.20 d (6.25 Hz); 2.29 dd (14.36 Hz; 6.25 Hz); 2.40 dd (7.10 Hz;14.36 Hz)
2-Hydroxyisobutyrate	1.31 s; 1.26 s
3-Hydroxyisovalerate	1.27 s; 2.36 s
3-Phenylpropionate	7.38 – 7.34 m; 7.32 – 7.29 m; 7.27 – 7.23 m; 2.91 – 2.85 m; 2.48 – 2.44 m
4-Aminobutyrate	3.03 – 3.06 m; 2.98 – 3.01 m; 2.28 – 2.30 m; 1.88 – 1.90 m
Acetate	1.90 s
Alanine	1.47 d (7.27 Hz); 3.80 – 3.74 m
Allantoin	4.12 s
Betaine	3.25 s; 3.87 – 3.89 m
Butyrate	2.15 – 2.11 m; 1.56 – 1.50 m; 0.90 – 0.86 m
Choline	4.08 – 4.04 m; 3.52 – 3.49 m; 3.19 s
Citrate	3.51 d (15.15 Hz); 2.65 d (15.15 Hz)
Creatine	3.02 s; 3.92 s
Creatine phosphate	3.03 s; 3.95 s
Creatinine	4.04 s; 3.04 - 3.03 m
Dimethyl sulfone	3.14 s
Dimethylamine	2.50 s
Formate	8.44 s
Galactarate	4.25 s; 3.94 s
Glucose	4.64 d (7.92 Hz); 3.88 dd (2.13 Hz;13.33 Hz); 3.84 – 3.50 m; 3.74 – 3.69 m; 3.52 dd (3.84 Hz;9.88 Hz); 3.49 m (9.88 Hz); 3.48 – 3.44 m
Glutamate	3.78 – 3.74 m; 2.36 – 2.88 m; 2.16 – 2.08 m; 2.08 – 2.00 m
Glutamine	3.78 – 3.75 m; 2.48 – 2.40 m; 2.18 – 2.10 m
Glycerol	3.78 – 3.90 m; 3.57 – 3.66 dd; 3.59 – 3.61 m; 3.38 – 3.47 dd
Glycine	3.50 s
Glycolate	3.94 s
Hippurate	7.84 – 7.80 m; 7.65 – 7.61 m; 7.56 – 7.52m; 3.96 – 3.93m
Histidine	7.88 s; 7.08 s; 4.00 – 3.96 m
Isobutyrate	2.42 – 2.31 m; 1.06 d (7.05 Hz)
Isoleucine	3.66 d (4.02 Hz); 1.00 d (6.96 Hz); 0.95 – 0.91 m
Lactate	4.10 m (6.98; 13.88); 1.32 d (6.98 Hz)
Leucine	1.76 – 1.64 m; 0.95 d (6.15 Hz); 0.94 d (6.15 Hz)
Mannose	5.20 - 5.22 d; 3.88 – 4.00 dd; 3.79 – 3.87 dd; 3.77 - 3.84 dd; 3.69 – 3.77 dd; 3.65 – 3.75 dd; 3.32 - 3.58 dt
Methanol	3.34 s
Methionine	2.65 – 2.61 m; 2.16 – 2.10 m; 2.12 s
Methylmalonate	3.14 – 3.18 m; 1.22 – 1.24 d
N-Nitrosodimethylamine	3.14 s; 3.82 s

Ornithine	3.55 – 3.58 m; 2.90 – 3.15 m; 1.89 – 1.99 m; 1.89 – 1.99 m; 1.77 – 1.84 m
Phenylalanine	7.41 m; 7.38 – 7.35 m; 7.33 – 7.30 m
Proline	4.12 – 4.09 m
Pyruvate	2.36 s
Sarcosine	3.60 s; 2.75 s
Succinate	2.39 m
Threonine	1.32 d (6.50 Hz); 3.58 d (5.15 Hz); 4.26 – 4.21 m
Tyrosine	7.20 – 7.17 m; 6.91 – 6.88 m
Valine	0.98 d (7.00 Hz); 1.03 d (7.05 Hz); 3.60 d (4.40 Hz)

s – simplet. d- dublet. m- multiplet.
